# Primary Cutaneous Gamma/Delta T-cell Lymphoma and Hemophagocytic Lymphohistiocytosis Associated With AIDS

**DOI:** 10.7759/cureus.10386

**Published:** 2020-09-11

**Authors:** Arjun C Khadilkar, Jacob J Adashek, Nicole D Riddle, Lubomir Sokol

**Affiliations:** 1 Internal Medicine, University of South Florida Morsani College of Medicine, Tampa, USA; 2 Pathology, Tampa General Hospital, Tampa, USA; 3 Hematology and Oncology, Moffitt Cancer Center, Tampa, USA

**Keywords:** aids, hlh, pctcl

## Abstract

Primary cutaneous gamma delta T-cell lymphoma (PCGD-TCL) is a rare lymphoma that makes up less than 1% of all cutaneous T-cell lymphomas. Patients with PCGD-TCL typically present with rapidly progressing plaques and ulceronecrotic nodules most frequently located on extremities without lymph node or bone marrow involvement. The overall prognosis is poor with a median overall survival of approximately 15 months. This case highlights a patient with concomitant PCGD-TCL, hemophagocytic lymphohistiocytosis, and human immunodeficiency virus-1-acquired immunodeficiency syndrome. There is a paucity of case reports describing PCGD-TCL and there are no evidence-based treatment recommendations. Further studies are needed to optimize strategies to treat patients with these diseases.

## Introduction

Human immunodeficiency virus-1 (HIV-1) increases the risk for developing various malignancies due to chronic antigenic stimulation of B-cells, cytokine dysregulation, and chronic inflammation [1,2]. Patients with HIV are predisposed to the development of opportunistic infections with oncogenic herpesviruses, such as Epstein-Barr virus (EBV) and human herpes virus 8 [3]. Epidemiological studies suggested that HIV infection increases the risk of developing non-Hodgkin’s lymphoma by approximately 60-200 times. Furthermore, patients with HIV are 15 times more likely to develop T-cell malignancies [4].

Accounting for less than 1% of all cutaneous T-cell lymphomas, primary cutaneous gamma/delta T-cell lymphoma (PCGD-TCL) is a rare lymphoma [5]. To differentiate PCGD-TCL from subcutaneous panniculitis like-T cell lymphoma (SPTL), it is important to note that the malignant CD8+ T cells express T-cell receptor (TCR) alpha/beta receptors in contrast to the gamma/delta receptors found on PCGD-TCL. A recent study identified germline bi-allelic loss-of-function mutations in the HAVCR2 gene in the majority of patients with SPTCL [6]. The HAVCR2 gene encodes the check point inhibitor T-cell immunoglobulin mucin-3 (TIM-3). The loss of TIM-3 expression on mature T cells is most probably driving oncogenesis with ultimate development of T cell lymphoma and chronic auto-inflammatory status resulting in hemophagocytic lymphohistiocytosis (HLH) [6].

Patients with PCGD-TCL often present with rapidly progressing plaques and ulceronecrotic nodules most frequently located on the extremities; typically, lymph nodes, spleen, and bone marrow are not involved at initial presentation [7]. Recently, Daniels et al. identified and characterized two molecular subtypes of PCGD-TCL; Vdelta-1 subtype classically involves the epidermis and dermis while Vdelta-2 represents the panniculitic form, which has been associated with a more aggressive clinical course and unfavorable prognosis. Unlike SPTCL, germinal mutations in the HAVCR2 gene have been shown to be absent in patients with PCGD-TCL [8]. Immunohistochemistry in PCGD-TCL reveals TCR gamma/delta+, CD3+, CD4-, CD8-, CD56+ and betaF1- immunophenotype. Malignant cells also characteristically express cytotoxic proteins such Granzyme B, TIA-1, and perforin [7,9,10]. 

HLH is generally divided into two major subtypes: primary and secondary. Primary or familial HLH manifests usually in children or young adults due to inherited defect of cytotoxic molecules such as perforin. Secondary HLH occurs in patients at high inflammatory states that are often related to ongoing viral infections, various malignancies, or autoimmune disorders [11]. The diagnosis of HLH is defined by having at least five of eight clinic-pathological criteria [12]. The pathology of HLH revolves around a hyper-inflammatory syndrome with unregulated T-lymphocyte and macrophage activation and decreased natural killer (NK) cell function [13]. There is a paucity of well described cases in the literature on PCGD-TCL and HLH.

The direct association between HIV and HLH is rare; typically, HLH develops in patients with chronic not well-controlled HIV or HIV-associated infections [14]. There are a limited number of case reports highlighting the association of HLH secondary to an acute HIV infection [15]. Herein, we present a patient with acquired immunodeficiency syndrome (AIDS) who was subsequently diagnosed with HLH and PCGD-TCL. To our knowledge, this represents the first case of a patient simultaneously diagnosed with these three conditions.

## Case presentation

**Figure 1:**
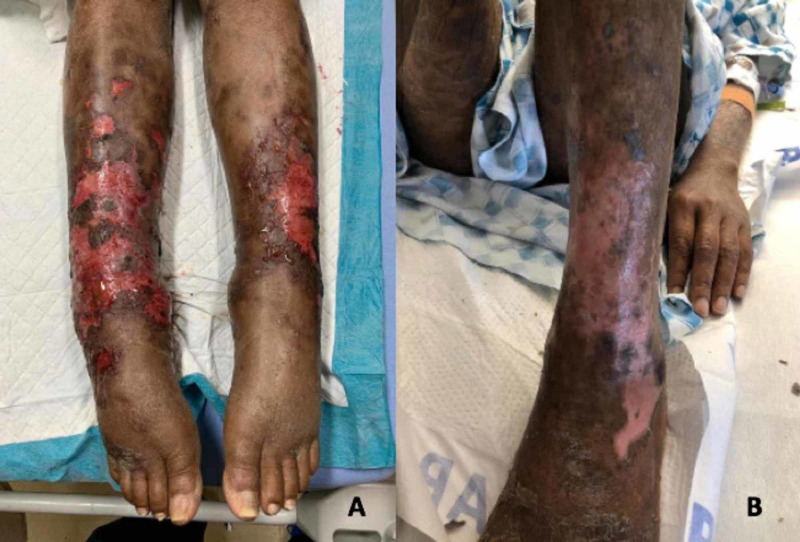
Progressing Plaques and Ulceronecrotic Nodules A: Lower extremity lesions upon initial presentation B: Lower extremity lesions after first cycle of cyclophosphamide, doxorubicin, etoposide, vincristine, prednisone (CHOEP)

A 53-year-old Haitian woman with known AIDS, CD4 count of 37, and HIV-1 (low viral load with <30 copies/mL), presented with a month of bilateral leg swelling associated with skin breakdown, serosanguinous discharge, and pain with ambulation (Figure 1). She had recently immigrated from Haiti and had significant lapses in HIV antiretroviral therapy along with medication access and compliance issues.

**Figure 2:**
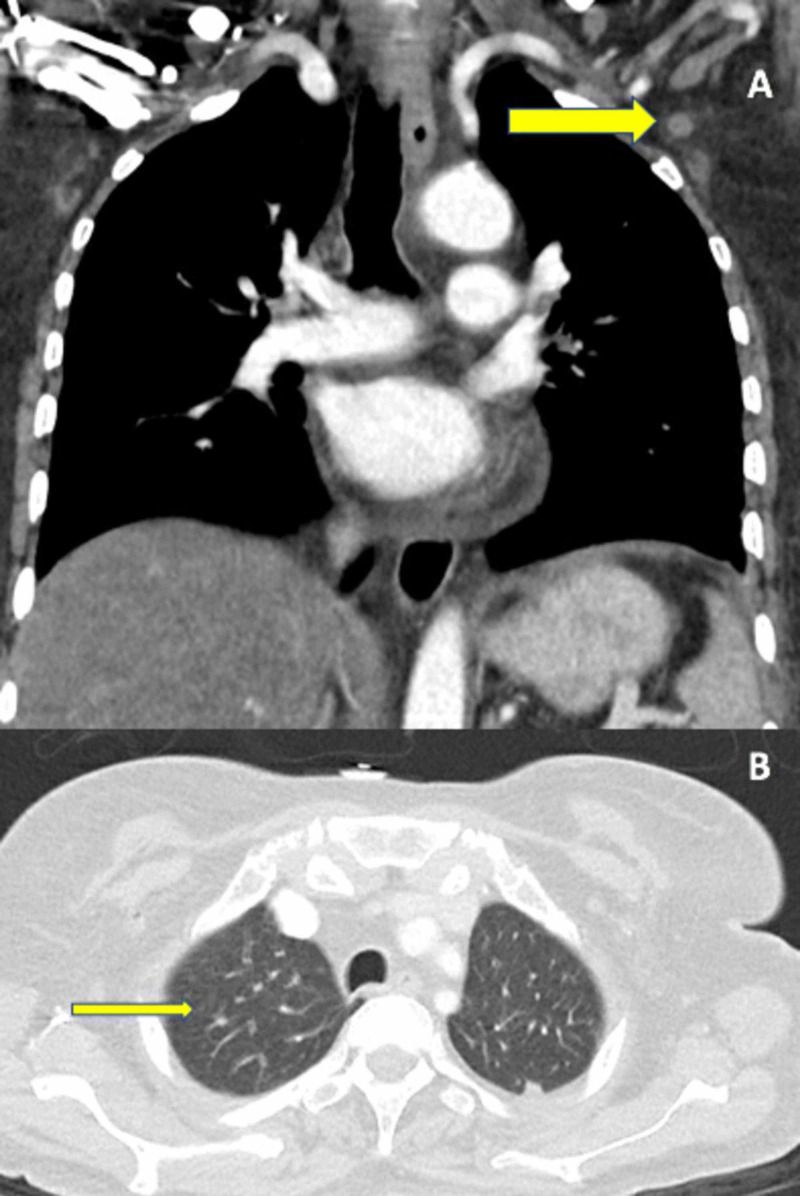
Computed Tomography (CT) Findings A: Mildly Prominent Axillary Lymph Nodes B: 5-mm Left Upper Lobe Pulmonary Nodule

On admission, there was concern for sepsis since she was found to be acidotic with a lactate of 3.3 mmol/L (reference: 0.5-1 mmol/L) in the setting of pancytopenia, most notably an absolute neutrophil count of 930 cells/mm3 (reference: 1,500 to 8,000/mm3). In light of these findings, she was started on broad-spectrum antibiotics with piperacillin-tazobactam and vancomycin, and underwent computed tomography (CT) scans of her chest, abdomen, and pelvis as recommended by the infectious disease specialists. The contrast CT scan (Figure 2) was significant for mildly prominent axillary lymph nodes and multiple pulmonary nodules. The origin of lymphadenopathy was presumed to be infectious.

To evaluate her for HLH, in the setting of HIV infection, ferritin (14,637 ng/mL, reference: 4.63-204 ng/mL), EBV PCR (1229 IU/mL, reference: negative), Interleukin-2 (IL-2) receptor (8280 pg/mL, reference: 175.3-858.2 pg/mL), triglycerides (240 mg/dL, reference: <150 mg/dL), and fibrinogen (48 mg/dL, reference: 190-500 ng/mL) were ordered. She additionally underwent a bone marrow biopsy, which showed erythroid hyperplasia, 1% myeloblasts, and evidence of histiocytes with hemophagocytosis without evidence of leukemia, lymphoma, granuloma, or metastatic carcinoma. Cytomegalovirus (CMV) polymerase chain reaction (PCR) plasma was undetected and EBV PCR plasma was positive with 1229 deoxyribonucleic acid (DNA) copies/mL. These findings resulted in the formal diagnosis of HLH based on clinical and laboratory studies.

**Figure 3:**
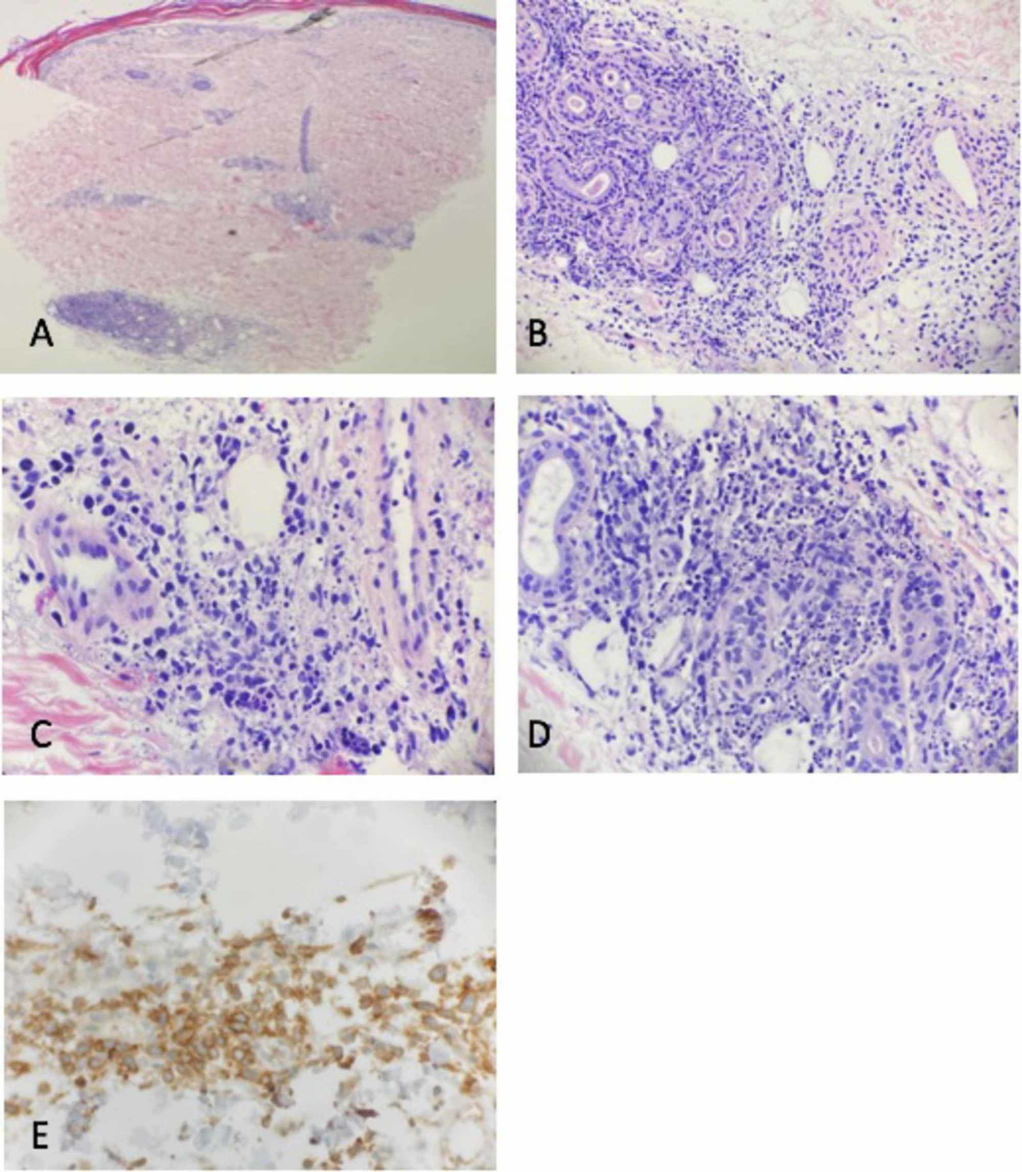
Histopathology of Primary Cutaneous Gamma Delta T-Cell Lymphoma (PCGD-TCL) Low power view demonstrating a perivascular and periadnexal infiltrate (A), Medium power view demonstrating a hyperchromatic atypical cellular infiltrate around adnexal glands and dermal vasculature (B), Higher power views demonstrating a marked amount of nuclear debris along with scattered large hyperchromatic atypical cells (C, D), CD3 immunostudy highlighting the T-Cell nature of the infiltrate (E)

To better evaluate her leg wounds, a punch biopsy was performed and histopathology with atypical lymphocytic infiltrate of T-cell origin; immunohistochemistry (IHC) was positive for CD2, CD3, weakly positive for CD5 and CD7 and negative for betaF1 consistent with PCGD-TCL (Figure 3). Further IHC investigation found atypical lymphocytes that were CD4 negative and CD8-double negative, and characterized by CD56 negative, Granzyme B negative, and TIA-1-positivity. In addition, CD20, CD30, CD34, and HHV-8 were negative. Polymerase chain reaction revealed positive clonality studies for T-cell receptor gamma and beta gene rearrangement and a further gamma/delta immunostain highlighted the atypical lymphocytes confirming the diagnosis of PCGD-TCL.

In contrast to more common cutaneous T cell lymphomas such as mycosis fungoides or CD30+ lymphoproliferative disorders, patients with primary cutaneous gamma delta T cell lymphoma have more aggressive disease and can benefit from induction treatment with combination chemotherapy regimens [16]. The patient was started on cyclophosphamide, doxorubicin, etoposide, vincristine, and prednisone (CHOEP) and after cycle three, she underwent a repeat contrast CT scan of abdomen and pelvis, which showed stable lymphadenopathy, unchanged from her initial imaging studies. However, she demonstrated significant improvements in her skin lesions with continued discoloration and flaking on her lower extremities. A repeat left thigh shave biopsy was performed, which showed no evidence of malignancy. She continued to have an elevated ferritin that rose from 3,851 ng/mL at discharge to 15,295 ng/mL approximately six months later. The increase in ferritin, in conjunction with her negative skin biopsy, prompted the concern for progressive HLH and she was initiated on dexamethasone according to HLH-94 and treatment protocol. Her performance status continued to decline, and she became acutely hypoxic where she was re-hospitalized and diagnosed with a right lower lobe pulmonary embolism (PE). Approximately one month after initiating treatment for her PE, and eight months after her initial diagnosis of PCGD-TCL, our patient passed away due to complications from her PE.

## Discussion

There are no randomized prospective clinical trials to determine the best therapy regimen for PCGD-TCL due to rarity of this disease. The median overall survival (OS) for PCGD-TCL is roughly 15 months [7,9] in contrast to eight months in the present case. However, it has been reported elsewhere that the patients with Vdelta-2 genotype and HLH have usually shorter survival [8]. There are no definitive guideline-directed treatments for PCGD-TCL, which include anthracycline-based chemotherapy, psoralen ultraviolet A light, and immunosuppressive therapies [11,17]. In addition, a small case series has recommended treatment with brentuximab vendotin, an antibody-drug conjugate consisting of an anti-CD-30 monoclonal antibody conjugated with anti-microtuble toxin monomethyl auristatin E, in patients with CD30+ PCGD-TCL. This report suggested that brentuximab vendotin, which was previously Food and Drug Administration (FDA) approved for therapy of mycosis fungoides, primary cutaneous anaplastic large cell lymphoma, and lymphomatoid papulosis, could be potentially expanded to PCGD-TCL. Although a sample size of only four patients, three patients had a positive response after four to six cycles of brentuximab vendotin and one patient had a minor response after two cycles [18]. This demonstrates the need for continued advancement for treatment regimens for PCGD-TCL.

In addition, there is no consensus recommendation for treatment of concomitant HLH and cutaneous TCLs. A majority of patients are treated according to HLH-94 or HLH-2004 protocols. Consolidation with allogenic hematopoietic stem cell transplant (alloHSCT) is believed to provide the most meaningful long-term benefit in patients with familial HLH or recurrent acquired HLH [19]. PCGD-TCL progression associated with chronic immunosuppression such as in patients with concomitant HIV infection may further lead to the failure of the host cytotoxic lymphocytes to eliminate activated histiocytes and propagate HLH development [20].

## Conclusions

Our patient had multiple risk factors that contributed to the development of HLH, including EBV and HIV infections and PCGD-TCL. The concomitant HLH and AIDS also portended survival disadvantages and led to her overall demise and poor prognosis. HLH can progress or relapse even though primary trigger has been eliminated. Further studies are warranted looking into optimal treatment strategies for these patients and to uncover additional insights into the association of these three disease processes to one another. Earlier diagnosis of these pathologies may lead to better patient care and improved clinical outcomes. 
